# Sea Surface Temperature Influence on Terrestrial Gross Primary Production along the Southern California Current

**DOI:** 10.1371/journal.pone.0125177

**Published:** 2015-04-29

**Authors:** Janet J. Reimer, Rodrigo Vargas, David Rivas, Gilberto Gaxiola-Castro, J. Martin Hernandez-Ayon, Ruben Lara-Lara

**Affiliations:** 1 Department of Plant and Soil Science, University of Delaware, Newark, DE, 19716, United States of America; 2 Departamento de Oceanografía Biológica, Centro de Investigación Científica y de Educación Superior de Ensenada (CICESE), Ensenada, Baja California, México; 3 Programa Mexicano del Carbono, Texcoco, Estado de México, México; 4 Instituto de Investigaciones Oceanológicas, Universidad Autónoma de Baja California, Ensenada, Baja California, México; University of Vigo, SPAIN

## Abstract

Some land and ocean processes are related through connections (and synoptic-scale teleconnections) to the atmosphere. Synoptic-scale atmospheric (El Niño/Southern Oscillation [ENSO], Pacific Decadal Oscillation [PDO], and North Atlantic Oscillation [NAO]) decadal cycles are known to influence the global terrestrial carbon cycle. Potentially, smaller scale land-ocean connections influenced by coastal upwelling (changes in sea surface temperature) may be important for local-to-regional water-limited ecosystems where plants may benefit from air moisture transported from the ocean to terrestrial ecosystems. Here we use satellite-derived observations to test potential connections between changes in sea surface temperature (SST) in regions with strong coastal upwelling and terrestrial gross primary production (GPP) across the Baja California Peninsula. This region is characterized by an arid/semiarid climate along the southern California Current. We found that SST was correlated with the fraction of photosynthetic active radiation (fPAR; as a proxy for GPP) with lags ranging from 0 to 5 months. In contrast ENSO was not as strongly related with fPAR as SST in these coastal ecosystems. Our results show the importance of local-scale changes in SST during upwelling events, to explain the variability in GPP in coastal, water-limited ecosystems. The response of GPP to SST was spatially-dependent: colder SST in the northern areas increased GPP (likely by influencing fog formation), while warmer SST at the southern areas was associated to higher GPP (as SST is in phase with precipitation patterns). Interannual trends in fPAR are also spatially variable along the Baja California Peninsula with increasing secular trends in subtropical regions, decreasing trends in the most arid region, and no trend in the semi-arid regions. These findings suggest that studies and ecosystem process based models should consider the lateral influence of local-scale ocean processes that could influence coastal ecosystem productivity.

## Introduction

Many land and ocean processes are linked through connections in the atmosphere. Synoptic-scale atmospheric events such as El Niño/Southern Oscillation (ENSO; 2 to 7 year cycles), Pacific Decadal Oscillation (PDO), and North Atlantic Oscillation (NAO; decadal cycles) are known to influence the global terrestrial carbon cycle [1,2]. ENSO and PDO are the predominant atmospheric phenomena which impact both marine and terrestrial ecosystems along the Pacific coast of North America [3]. For example, the net ecosystem production in Douglas-fir and conifer forests along the Pacific coast increases (or decreases) due to strong ENSO leading modes and air temperature anomalies [4,5]. Therefore, evidence suggests that connective processes influence regional-scale gross primary production (GPP) in coastal ecosystems. It is unknown, however, how smaller scale processes, including those of marine origin (e.g., local upwelling events) could influence local-to-regional variability of GPP.

In arid coastal climates along eastern boundary currents (such as the Baja California Peninsula and the coastal regions of Chile and Peru) water vapor of marine origin provides moisture for local vegetation as well as intensifies the warming of the coastal landmass creating a positive feedback [6,7]. Terrestrial warming could promote an ocean-land thermal contrast and greater ocean-land atmospheric pressure gradients which, in turn, could influence upwelling-favorable winds. Furthermore, atmospheric relative humidity positively influences GPP and soil CO_2_ efflux variability in coastal environments across semi-arid regions [8–10]. It is well known that sea surface temperature (SST) has an effect on the regional atmospheric relative humidity: as cold water comes to the surface, water vapor forms because the air is dry or because cooler air is trapped below a warmer air mass thus forming water vapor (i.e., fog); known as the “marine layer” [11]. Another way in which water vapor content increases over the surface ocean is via evaporation when the air is warmer than the SST [12]. Over the last several decades, long-term intensification of upwelling due to intensification of offshore winds has been observed in the major upwelling systems off Peru and the Californias due to large-scale ocean-atmosphere heat exchange [13,14]. Synoptic-scale (basin-wide and greater) model simulations of upwelling, however, predict a contemporary weakening of global upwelling [15], while small scale models (in the spatial domain: tens to hundreds of kilometers) have shown intensification due to wind when applied to the California Current System [16]. Therefore, it is important to determine how the future of coastal upwelling regions will be affected by climate variability driven by wind [17], and by extension how these regions will affect their adjacent coastal land masses; specifically the ecophysiological responses of arid ecosystems.

In the coastal zone of the southwestern United States and northwestern Mexico the El Niño phase of ENSO presents as increased SST, surface ocean stratification (relaxation of upwelling; [18]), and precipitation [19], as well as decreased wind velocities [20–22]. The reduction in upwelling and increased stratification (due to decreased wind velocity) also leads to declined marine gross primary production [23]. The Baja California Peninsula is characterized by various semiarid climate regimes including Mediterranean (northwest), desert (central), and subtropical (extreme south), with vegetation in all regions adapted to drought conditions (La Niña phase). This type of vegetation may rapidly grow during wet conditions (El Niño phase) [24]. The patterns of GPP and the relationships to changes in SST (i.e., localized ocean-land connection and teleconnections) across gradients of vegetation types and climates along the Baja California Peninsula are presently unclear. Therefore, the Baja California Peninsula and the southern California Current boundary present an excellent case-study to test for ocean-land connections across semi-arid climates and vegetation types.

The objectives of this study are: a) to determine if there is an ocean-land connection between SST and GPP, and ENSO and GPP along the latitudinal gradient of the Baja California Peninsula; and b) determine which processes, SST (representing local-scale processes) or ENSO, (representing synoptic-scale processes) has higher influence on GPP variability. It should be pointed out that herein we distinguish differences between teleconnection (synoptic-scale) and ocean-land connection (scale of up to ~70 km; offshore region to the terrestrial site farthest from the coast representing local-scale processes). For example, teleconnective processes between Pacific Ocean evaporation and continental (North America) precipitation/transpiration are known to occur [25,26]. Therefore, on smaller spatio-temporal scales, we hypothesize that coastal SST influences GPP along (north/south) and across (west to east) the Baja California Peninsula as these terrestrial arid ecosystems could be influenced by water vapor input from the ocean [5,8–10] since coastal upwelling is driven by meso-scale [17,27] and synoptic-scale atmospheric pressure gradients [28]. We expect that GPP in coastal ecosystems will be coupled at short time-scales (i.e., weeks to months) with changes in SST, and a lower variability of GPP may be explained by synoptic-scale atmospheric forcing such as ENSO. Since the Baja California Peninsula is over 1,200 km long, we are able to examine local scale ocean-land connection and interannual variability across a climate/vegetation gradient to test their responses to similar connective and teleconnective forcing.

### Regional setting

The Baja California Peninsula is bordered by the Pacific Ocean to the west and by the Gulf of California to the east. The California Current (CC), a cold eastern boundary current located off the coast of the Californias (distance varies by season; [29]) on top of a colder, more dense, nutrient-rich deep water mass which is upwelled to the surface when strong, persistent winds prevail from the north-northwest. The cold, nutrient-rich upwelled waters fuel the high levels of ocean productivity along the peninsula [23] and are known to intensify fog development [16] that could influence GPP [10]. The “upwelling season” (when upwelling is strongest and most persistent) is typically from March through August, though upwelling does occur year round. Upwelling along Baja California is strongest off the capes and points [27] and is the primary selection criterion for the regions we examine herein. Finally, the oceanic region to the west of the Californias is known to supply atmospheric moisture to the hydrologic cycle of these arid to semi-arid ecosystems [25].

The climate gradient along the peninsula is such that the southern portion is affected by tropical cyclones in the late summer-early fall months (typically August through October) with the dry season occurring from November to late June [30]. Whereas the northern portion is characterized by winter rains, year round subtropical high pressure, and maritime polar air masses [31]. The peninsula is mountainous in the center with some narrow coastal plains on the Pacific coast. The mountains between the Pacific Ocean and Gulf of California coasts block the clouds and moist air associated with the air masses coming off the Pacific; therefore the western side of the Baja California Peninsula has greater relative humidity than the eastern [32].

#### Transect description

Three transects (from offshore to the middle of the peninsula) were chosen along the Pacific coast off the Baja California Peninsula (from north to south): Punta Colonet (PC), Punta Abreojos (PA), and Bahia Magdalena (BM; Table 1). Transects were chosen based on their proximity to stronger upwelling regions (points and capes), different climates, vegetation, and the lack of anthropogenic influence (Table 1). Each transect consists of 2 or 3 sites, which are located at sea level or on upward slopes (<1,000 meters above sea level).

**Table 1:** General location and physical description of the terrestrial transects and the individual characteristics of the MODIS cutoffs (sites).

Site (north to south)	Latitude/Longitude	Approximate distance to coast (km)*	Vegetation type (center pixel)	Climate
**PC1**	30.97849°N, -116.30985°W	5.0	Closed shrubland	Semi-arid
**PC2**	30.97849°N, -116.11176°W	13	Closed shrubland	Semi-arid
**PC3**	30.97849°N, -116.30985°W	50	Open shrubland	Semi-arid
**PA1**	26.96033°N, -113.70618°W	12	Open shrubland	Arid
**PA2**	26.96033°N, -112.74719°W	57	Open shrubland	Arid
**MB1**	24.76429°N, -111.85455°W	29	Open shrubland	Sub-tropical
**MB2**	24.76429°N, -111.59912°W	57	Open shrubland	Sub-tropical
**MB3**	24.76429°N, -111.09375°W	60^†^	Open shrubland	Sub-tropical

*Approximate distance to the ocean coast, not a bay, from the center pixel of the cutoff.

^†^BM3 is 60 km from the coast along a southwest oriented transect, but approximately 95 km along the line of latitude.

The Punta Colonet transect (sites PC1, PC2, and PC3) is the region farthest north along the peninsula studied in this work and extends up to 68 km inland to the base of the San Pedro Martir mountain range. The vegetation types were: closed shrubland (PC1 and PC2), and open shrubland (PC3; Table 1). The Punta Abreojos transect (PA1 and PA2) is in one of the driest areas of the Baja California Peninsula and is also the driest region analyzed in this study. At this transect we were only able to select two sites (i.e., NASA’s Moderate Resolution Imaging Spectroradiometer [MODIS] cutoffs) due to the inland oasis (subterranean water source) at San Ignacio and the location of San Ignacio Lagoon, which would likely influence water vapor content thus masking the effects of upwelling. Both sites are open shrubland (Table 1). The Bahia Magdalena (BM1, BM2, and BM3) transect extends approximately 85 km inland. All three sites are open shrubland (Table 1) and this is the wettest region (sub-tropical) in this study. The marine portion of the transect starts outside of the bay and stretches across the coastal zone.

## Methods

SST has been used as an indicator of coupled ocean-atmosphere variability to detect cold temperatures which may indicate the occurrence of wind driven coastal upwelling [33]. In this case we use changes in SST as a proxy for moisture transport due to upwelling events. Monthly composite SST observations were obtained from MODIS through the NASA Giovanni server (http://gdata1.sci.gsfc.nasa.gov/daac-bin/G3/gui.cgi?instance_id=ocean_month). We used the 4 km^2^ resolution night SST product to avoid the influence of solar heating of the ocean surface skin layer. Since upwelling is a process in which the wind-ocean interaction may extend for roughly one day to several days, using the night product will not filter out the effect of upwelling. The time series of SST is averaged over the spatial domain of the offshore region for each transect: ~4 km (north/south; the finest resolution of the data) by up to ~100 km (east/west). The offshore length of the marine transect incorporates the California Current ([26], and references therein.) Temporally, SST observations are available and overlap with terrestrial products for complete years from 2003 through 2013.

We used the fraction of photosynthetically active radiation (fPAR) MODIS Land Product (MOD15A2) for cutoffs of 25 km^2^ at each transect from 2003 through 2013 (2 to 3 inland cutoffs per transect), with the mean value for fPAR reported for the center pixel. fPAR is a unitless ratio (m^2^ m^-2^) representing the radiation (0.4 to 0.7 μm) which is absorbed by vegetation [34] and can be used as a proxy for GPP temporal patterns. The amount of radiation absorbed gives insights into the amount GPP at each site as it is the energy which is absorbed by the plant for photosynthesis and growth. Furthermore, fPAR in combination with daily incident radiation and the light use efficiency of the plant canopy are the key variables to calculate GPP for the MOD17 product [35]. fPAR and GPP observations are derived from MODIS products generated with Collection 5 from the Oak Ridge National Laboratory Distributed Active Archive Center (ORNL DAAC, 2009). Details about preparation of subsets including MODIS data reprocessing, methods, and formats are available from http://daac.ornl.gov/MODIS/. Along each transect the cutoffs for MODIS fPAR data are referred to as “sites”; the sites are treated separately for statistical analyses, and then general conclusions are made in terms of all the sites that make up a transect. MODIS fPAR was analyzed for years 2000–2013 and MODIS GPP for years 2000–2010. GPP observations are combined with fPAR for temporal analyses due to the larger extent of available data at the region of interest, but as described earlier, it is a proxy for GPP.

An initial analysis of data using eight-day temporal resolution showed that SST influences fPAR on a time scale of 1 and 2 months (cross correlation analysis, results not shown). Therefore, we continue with data analyses using monthly means for SST and fPAR for statistical analyses, and GPP to examine spatial and temporal variation as the annual sum of GPP. Comparisons of yearly sums for GPP were calculated by adding the eight-day mean value of the MODIS product (MOD17) with the calculated standard error. We used the Multivariate ENSO Index (MEI) for the analysis of the synoptic-scale teleconnection between oscillations in atmospheric conditions and the response in fPAR (data available from: http://www.esrl.noaa.gov/psd/enso/mei/table.html). The temporal resolution of this index is also one month and therefore a period of two months is the lowest frequency for which we are able to resolve synoptic scale variability as indicated by the Nyquist frequency (i.e., the lowest sampling frequency is equal to two times the change in the time step [36]).

We use a linear least squares fit analysis of the eight-day time series to determine the change over time in fPAR for the 11 years of the study period. The least squares approach minimizes the noise in data sets. Since we are analyzing 11 years of observations, we caution that the change over time (i.e., trend or slope) only represents relatively short term secular change. Cross correlation analysis is used to determine the time scale on which connections are occurring (all with *p* values set an α of 0.05). Linear regression analysis was applied to determine the percentage of variability in fPAR that may be explained by monthly composite SST changes and MEI.

## Results

**Fig 1:**
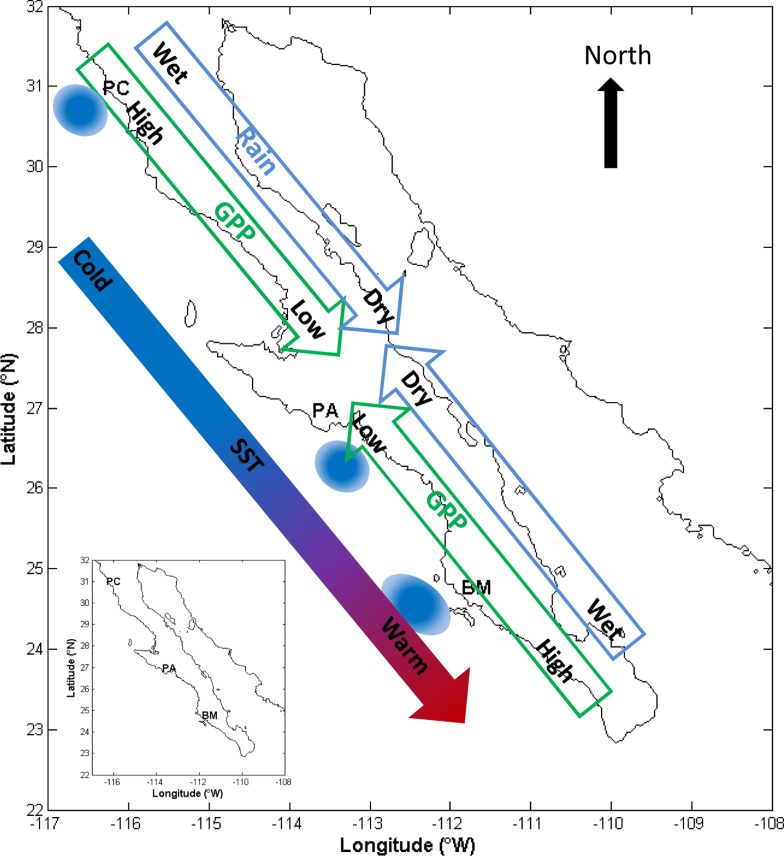
A conceptual summary of precipitation, SST, and fPAR (GPP) along the Baja California Peninsula. The location of the transects are denoted by their abbreviations. SST is colder in the north (denoted by the blue color) and warmer in the south. The California Current roughly parallels the coast line, flowing north to south. The upwelling zones mentioned in this work are denoted by blue ovals. fPAR is higher in the extreme north and south (green color) and lower in the middle of the peninsula (brown color). Finally, precipitation is also higher in the northern and southern extremes and lower in the center of the peninsula (denoted by the fading blue colored arrow).

**Fig 2:**
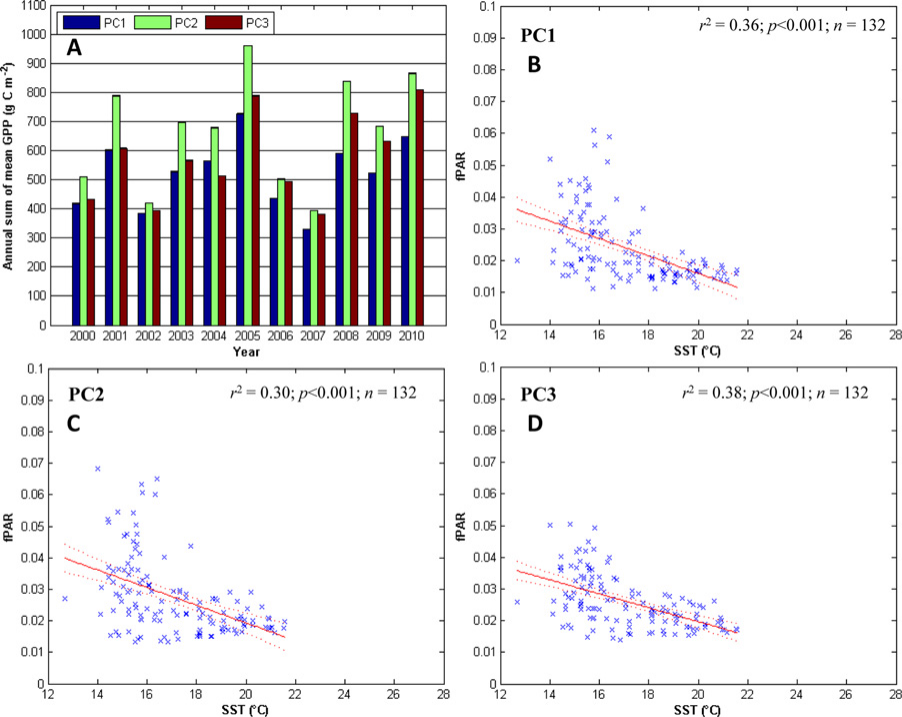
GPP and fPAR along the Punta Colonet transect. The Punta Colonet (PC) transect, clockwise from the top-left: A) the annual sum of mean GPP for all the sites along each transect. In each group of years the first bar from the left is the coastal site with each sequential bar representing the next site to the east. The error bars (standard error) are too short to be seen on the graphic. Panels B, C, and D) linear regression analysis for the SST (predictor) and fPAR (response) for PC1, PC2, and PC3, respectively.

**Fig 3:**
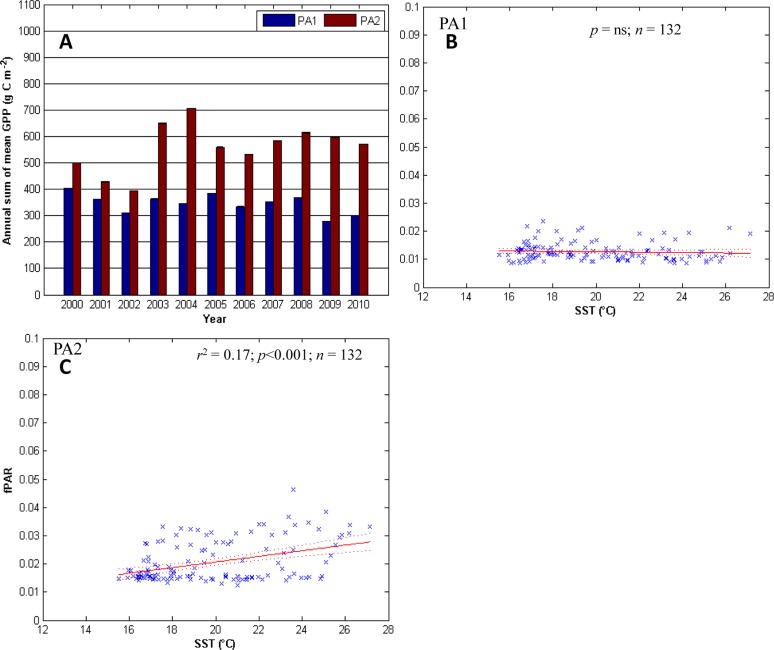
GPP and fPAR along the Punta Abreojos transect. The Punta Abreojos (PA) transect, clockwise from the top-left: A) the annual sum of mean GPP for all the sites along each transect. In each group of years the first bar from the left is the coastal site with each sequential bar representing the next site to the east. The error bars (standard error) are too short to be seen on the graphic. Panels B and C) linear regression analysis for the SST (predictor) and fPAR (response) for PA1, and PA3, respectively.

**Fig 4:**
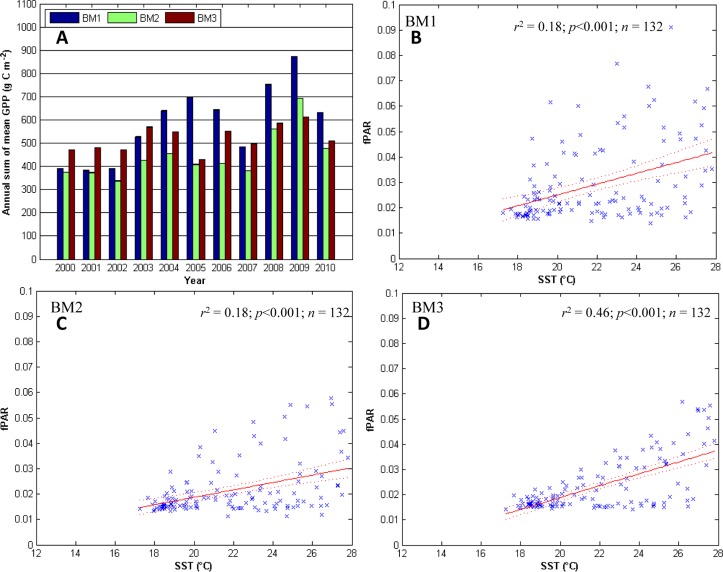
GPP and fPAR along the Bahia Magdalena transect. The Bahia Magdalena (BM) transect, clockwise from the top-left: A) the annual sum of mean GPP for all the sites along each transect. In each group of years the first bar from the left is the coastal site with each sequential bar representing the next site to the east. The error bars (standard error) are too short to be seen on the graphic. Panels B, C, and D) linear regression analysis for the SST (predictor) and fPAR (response) for BM1, BM2, and BM3, respectively.

Results are organized from north to south along the peninsula in order to emphasize the spatial differences between transects (Fig 1). Along each transect we focus on a synthesis of the overall characteristics, rather than a detailed comparison of site specific differences. We did not observe statistically significant temporal trends for SST for any transect. Thus, we find no evidence of temporal changes in SST along the CC. We do, however, find evidence of changes in fPAR over time with the trends varying in magnitude and direction (increase at the subtropical site, decreasing in the middle of the peninsula, and no trend at the two sites closest to the coast in the semi-arid northern site). All trends reported herein are significantly different than zero (95% confidence intervals [CI]) do not overlap with zero) with results reported in Table 2. Regression analyses for all sites were repeated omitting potential outliers. We could not define outliers based on typical statistical ranges (i.e., ± 3 standard deviations), as all of the data fell within this range due to the large variance in the distribution of the data. Therefore, we specifically omitted three specific data points at PC1, PC2, and PC3 (with SST ~13°C; Fig 2B, 2C and 2D), none at PA (Fig 3B and 3C), and at BM1 (fPAR ~0.09; Fig 4B) that appeared to potentially alter the results of the linear regression. Our results show that during all instances the repeat analyses were not statistically different from the initial analyses; therefore all results are presented using the former approach.

**Table 2:** Results of trend, cross correlations analyses for fPAR-MEI, fPAR-SST, and mean fPAR for the time series.

Site	Trend y^-1^	Slope	95% CI(±)		*p*-value of trend	SST-fPAR	MEI-fPAR	Mean fPAR (*x* 10^-2^)
**PC1**	—	—	—	ns		*r =* -0.52; 1 months*	*r =* 0.18; 9 months	2.4 ± 1.1
**PC2**	—	—	—	ns		*r =* -0.48; 2 months	ns	2.7 ± 1.2
**PC3**	0.0020	5.6 *x*10^-6^	1.1 *x*10^-5^	< 0.05		*r =* -0.52; 2 months	*r =* -0.21; 4 months	2.6 ± 0.8
**PA1**	-0.0016	-4.3 *x*10^-6^	8.7 *x*10^-6^	< 0.001		*r =* 0.35; 5 months	*r =* 0.28; 5 months	1.3 ± 0.3
**PA2**	-0.0023	-6.4 *x*10^-6^	1.3 *x*10^-5^	< 0.01		*r =* 0.62; 3 months	*r =* 0.28; 3 months	2.1 ± 0.7
**BM1**	0.0060	1.7 *x*10^-5^	7.1 *x*10^-3^	< 0.001		*r =* 0.70; 3 months	*r =* -0.34; 11 months	2.9 ± 1.5
**BM2**	0.0090	2.4 *x*10^-5^	4.4 *x*10^-3^	< 0.001		*r =* 0.64; 3 months	*r =* -0.44; 12 months	2.1 ± 1.0
**BM3**	—	—	—	ns		*r =* 0.80; 2 months	*r =* 0.22; 4 months	2.3 ± 1.1

All results are significant at *p*<0.05 or better unless otherwise indicated by “ns”. The time lags for cross correlation analysis are expressed as up to the number of months that the relationship exists. A negative correlation signifies that as one variable increases the other decreases and vice versa. Trend results for SST are not reported in this table since they are not significant. Trends for units per year for fPAR calculated from the slope of the least squares fit the respective confidence intervals are the values of the upper minus the lower bounds adjust to per year values. All analyses have an *n* = 132 (monthly means over the 11 years of the study), except the mean, which has an *n* = 506 (8-day resolution over 11 years).

*This result is interpreted as fPar is in phase with SST as we can only resolve variability at two times the frequency of the time step.

### Punta Colonet transect

Annual mean sum GPP was the highest of all transects with a maximum of ~960 g C m^-2^ y^-1^ at PC2 in 2005 (Fig 2A), and in general was highest at PC2. SST explains 30 to 38% of the variability in fPAR along this transect (Fig 2B to 2D). The slope of the best fit line for the relationship between fPAR and SST is significantly (*p* < 0.001) negative for all three sites (Fig 2B–2D). Using cross-correlation analysis we found that fPAR correlated with SST on a time scale of up to 1 month at PC1 and up to 2 months at PC2 and PC3 (Table 2). Following the Nyquist theorem, since we are only able to resolve variability at a frequency two times the time step, this one month time lag could be interpreted as SST and fPAR are in phase with each other. The MEI correlated with fPAR at PC3 (*p* < 0.05; n = 132) with a lag of up to five months (Table 2), however, the relationship was not significant at the other two sites along the transect. ENSO only explains up to 3.9% of the variability in fPAR at PC3, however, this relationship is not significant at the other two sites (Table 3). The spatial variability of GPP for this transect shows a general increase away from the coast (Fig 2). There is only a significant trend (*p* < 0.05) in fPAR at PC3, which was increasing at a rate of 0.002 units per year over the 11 years of the time series (Table 2).

**Table 3:** Results of linear regression analysis for determining the variability in fPAR explained by SST and MEI.

Site	fPAR-SST	fPAR-MEI
**PC1**	*r* ^2^ = 0.36; *p*<0.001	ns
**PC2**	*r* ^2^ = 0.30; *p*<0.001	ns
**PC3**	*r* ^2^ = 0.38; *p*<0.001	*r* ^2^ = 0.039; *p*<0.05
**PA1**	ns	*r* ^2^ = 0.035; *p*<0.05
**PA2**	*r* ^2^ = 0.17; *p*<0.001	*r* ^2^ = 0.068; *p*<0.05
**BM1**	*r* ^2^ = 0.18; *p*<0.001	*r* ^2^ = 0.037; *p*<0.05
**BM2**	*r* ^2^ = 0.18; *p*<0.001	ns
**BM3**	*r* ^2^ = 0.46; *p*<0.001	ns

“ns” denotes a non-significant relationship.

### Punta Abreojos transect

In general, GPP is higher away from the coast, with the maximum annual GPP at PA2 in 2004 (~700 g C m^-2^ y^-1^; Fig 3A). SST explains up to 17% of the variability in fPAR at PC2, however, this relationship is not significant at PC1 (Fig 3B to 3C). This transect has the longest lag times for the response of fPAR to SST: up to 5 months at PA1 and up to 3 months at PA2 (Table 2). fPAR at these sites lagged behind the MEI by 5 months at the PA1 and 3 months at PA2 (Table 2) with ENSO explaining up to 3.5 and 6.8% of the variability at PA1 and PA2, respectively (Table 3). There are significant (*p* < 0.01) decreasing trends in fPAR of -0.0016 and -0.0023 units per year at PA1 and PA2, respectively (Table 2).

### Magdalena Bay transect

Contrary to the other sites, at the beginning of the period studied, GPP was higher at the site farthest from the coast, and then became higher at the coast and lower inland in 2004. The maximum GPP along this transect was at BM1 in 2009 (~880 g C m^-2^ y^-1^; Fig 4A). SST explains 18% of the variability in fPAR at BM1 and BM2 and up to 46% at BM3 (Table 3; Fig 4B–4D). fPAR responded to SST on a time scale of up to 3 months at BM1 and BM2 and up to 2 months at BM3 (Table 2). fPAR lagged up to 11 and 12 months behind the MEI at BM1 and BM2, respectively, however, only 4 months at BM3 (Table 2). ENSO explains up to 3.7% of the variability in fPAR at BM1 but is not a significant source of variability at the other two sites. There are significant increasing trends in fPAR at BM1 and BM2 (0.0060 and 0.0090 units per year, respectively); however, the trend is not significant at BM3 (Table 2).

## Discussion

The results support our hypothesis that SST is a greater influence on fPAR (as a proxy for GPP) than the MEI (as a measure of ENSO) along the Baja California Peninsula, and that SST affects fPAR on a shorter time scale than ENSO. The ocean-land connection and teleconnection between SST and fPAR and MEI and fPAR are identified using the combined results of linear regression (i.e., influence of SST on variability of fPAR) and cross correlation (i.e., time lag of the maximum independent correlation between a change in SST/MEI and fPAR). Although *r* ^2^-values for the linear regressions are relatively low (do not explain much of the variability), quadratic fits resulted in even lower *r* ^2^-values (results not shown). We recognize that non-linear effects are likely to be present in ocean-land interactions, but, we interpret our results as a first estimation of the potential influence of changes in SST on GPP in arid and semi-arid climates that have been understudied.

The annual sum of mean GPP is highest in the northern portion of the peninsula with a decrease in the Central Desert region, and then an increase again in the southern portion in agreement with the changing climate regimes (i.e., semi-arid, arid, and sub-tropical; Fig 1). ENSO influence on GPP may increase away from the coast in the north (PC transect) and south along the coast (PA and BM transects). Herein we discuss the implication of the results in terms of potential weather variability.

### Relationships and trends in SST and fPAR

The PC transect is located at one of the most intense coastal upwelling regions along northern Baja California [27]. Up to 38% of the variability in fPAR along this transect can be explained by the changes in SST, with time lags increasing away from the coast of up to 2 months. Higher fPAR is associated to colder SST’s and is consistent with findings that water vapor enters the atmosphere due to condensation (cold marine layer influence; [37]). Water vapor is then an important source of moisture for GPP in this region [6,7]. Previous studies of the CC region have shown that wind-driven upwelling is intensifying [16], potentially decreasing mean monthly SST. The relationship between SST and fPAR (higher GPP values at lower SST) along the PC transects suggests that if there were a long term SST decrease due to intensified upwelling, then GPP would likely increase. Another previous study, however, which modeled smaller spatial scales found that a portion of the CC is in fact warming [14]. If this were the case, then GPP would decrease with the increased SST. We did not, however, find evidence of a significant secular trend in monthly composite SST over the 11 years studied at this site (Table 2). A climate change scenario in which terrestrial temperatures increase could enhance the thermal differential between the coastal ocean and the land surface, therefore increasing local winds [17]. Increased local winds may have an effect on localized upwelling, SST, and therefore GPP. In this region if upwelling intensifies then the predicted response would be an increase in GPP. Over the 11 years of our study period, we did find a significant increase in fPAR at PC3 (consistent with a potential decreasing trend in SST) where the greatest amount of variability is explained by SST.

The ocean-land connection between SST and fPAR along the PA transect showed the weakest relationship among the three transects and does not even appear to exist at PA1. Of all the sites, PA1 is the only site where fPAR and SST are not significantly related. GPP (and fPAR) was lowest at PA1 (Fig 3A and Table 2); since this is the most arid transect we analyzed and is open shrubland, a possible explanation for the lack of a significant relationship between fPAR and SST may be low fPAR values due to scarce vegetation (and consequently low GPP). This site has little vegetation due to the arid climate of the region, and what little vegetation there is independent of the changes in SST despite the potential effects of moisture in the air or the added moisture to the air is insufficient to have a significant effect on the vegetation. Therefore we will not explore the results at PA1 further and due to the characteristics of PA2, we include this site in the discussion of the BM transect.

Along the BM transect and PA2, as SST increased fPAR increased. SST explains up to 17% of the variability in fPAR at PA2 and is correlated to SST on a scale of up to 3 months. A similar scenario is also occurring along the BM transect, where higher fPAR is also related to higher SST with similar time lag and variability explained by SST (Tables 2 and 3). The exception, however, is at BM3, where 46% of the variability in fPAR is explained by SST and the lag time is shorter at this site (up to 2 months). The relationship of higher fPAR to higher SST is likely due to the fact that evaporation, rather than condensation (as along the PC transect), is responsible for the addition of water vapor to the atmosphere [12]. For example, higher SST contributes a greater amount water vapor to the troposphere [38]. For the climate change scenario which predicts a warming of the CC [14], increased SST in the sub-tropical region could contribute to increased GPP. Over the time period analyzed using monthly composite observations we do not find evidence of a change in SST over time (Table 2). We do, however, observe increasing significant trends in fPAR at PA1, PA2, BM1, and BM2 which could be caused, at least in part, due to a change in SST that we are unable to resolve at this time; therefore, longer time series are required to explain these trends. Along the BM transect and at PA2 we find that changes in SST are likely an important source of moisture to the atmosphere and therefore to vegetation. This explanation is supported with several studies that have shown that water vapor content is influenced by changes in SST [11,37] and that moisture transported in the atmosphere is important for plant growth [39]. Then, we assume that the potential moisture added to the atmosphere along the southern CC is likely affecting GPP along Baja California as seen through relationships with SST. Unfortunately, observations for precipitation do not presently overlap with the MOD15A2 fPAR product in this region due to the remoteness and the lack of a robust monitoring network.

### Synoptic-scale variability

Our results also support the hypothesis that synoptic-scale ENSO variability is less important than local-scale SST changes for fPAR variability. Annually, the MEI explains less than 7% (Table 2) of the variability in fPAR along the Baja California Peninsula, with the lag time lag increasing north to south (Table 1). fPAR at coastal sites in warmer SST regions (BM and PA) are significantly related to the MEI, whereas in the colder SST region (PC) the site farthest from the coast is the only site with a significant relationship (Table 2). The lack of a synoptic scale relationship for ENSO with fPAR is likely due to the fact that one of the characteristics of arid and semi-arid plants is that they are resistant to drought, and when there are sporadic rains they store the water rather than use it for growth [40]. Therefore, even during wetter years caused by the El Niño phase ENSO, local sources of variability such as water vapor content may be a more important influence on fPAR variability than synoptic-scale processes. This may also be evident in that the time lags time between fPAR and ENSO were shorter away from the coast along all the transects, which suggests that these sites are more sensitive to synoptic-scale variability than sites in closer proximity to coastal sources of moisture.

## Synthesis and Conclusions

We find that an ocean-land connection between SST and fPAR exists along the Baja California Peninsula but the influence varies north/south as well as east/west. These results are in general agreement with synoptic-scale studies that have identified the eastern Pacific as a water vapor source for North America [25,26], as it is likely this water vapor that is critical for plant growth in water-limited ecosystems. SST affected fPAR on a time scale shorter than the MEI. In fact, in some cases ENSO was not a significant source of variability in fPAR. Furthermore, we find that the time scale on which the ocean-land connection exists (from approximately zero, or in phase, to three months) also varies with latitude and distance from the coast. Fig 1 illustrates a summary of SST, precipitation, and fPAR and GPP along Baja California. While these spatial gradients may be specific to the Baja California Peninsula, it is likely that similar conditions may exist along the Humbolt Current where an arid climate is bordered by a cold upwelling region similar to the CC [41]. These findings are relevant because the influence of local-scale ocean processes are generally overlooked when attempting to understand ecophysiological processes in coastal ecosystems [8–10]. Furthermore, ecosystem process models fail to accurately represent ecosystem fluxes in water limited ecosystems [42] and therefore the lateral influence of local-scale ocean processes could influence water-limited coastal ecosystem productivity.

It should be pointed out that the SST reported in the present study, averaged over spatial and temporal scales, highlights monthly coastal changes in temperature (upwelling). Since the SST-fPAR relationship is significant at monthly time scales, the varying time scales of upwelling are best represented in this work as monthly mean SST, rather than at shorter time scales. This also takes into account the lag time between moisture entering the atmosphere and the response in fPAR (photosynthetic growth). Regional upwelling is a meso-scale process, however, due to local thermal land-ocean gradients, it may also be driven by smaller-scale processes (offshore thermal winds), and will not necessarily reflect synoptic-scale events [27] such as ENSO. This result suggests that even if there is an occurrence of more frequent drought due to the La Niña phase of ENSO, if upwelling in the north (semi-arid region) does not relax, then the vegetation along the PC transect will not be greatly affected. If, however, upwelling does relax, the opposite effect will occur. While in the arid and sub-tropical regions cold SST due to intensified upwelling could contribute to a decrease in GPP. Future research should include determining air parcel trajectories in order to determine which regions are most greatly affected by the onshore transport of water vapor due to SST. This could be achieved using Lagrangian techniques as they are useful for identifying the typical paths of advective moisture in both lower and higher levels of the atmosphere [43].

We conclude that a) an ocean-land connection exists between SST (as an indicator of both upwelling and relaxed conditions) and fPAR (as a proxy for GPP) in different climate regions of the Baja California Peninsula and is a greater influence on fPAR variability than synoptic-scale mechanisms such as ENSO; b) colder SST in the northern portion of the peninsula contributes to increased fPAR values, while warmer SST contributes to increased GPP in the southern portion; and c) GPP is likely indirectly affected by the influence of SST on water vapor contributed to the atmosphere, and there is a clear distinction in the response between northern (decreased SST, increased fPAR) and southern (increased SST, increased fPAR) portions of the peninsula, as well as east/west across the peninsula. A complete understanding of the controls on the regional-to-global carbon cycle must include a local-scale understanding of the sources and scales of variability and their respective contributions.
